# Anthocyanins: From the Field to the Antioxidants in the Body

**DOI:** 10.3390/antiox9090819

**Published:** 2020-09-02

**Authors:** Vidmantas Bendokas, Vidmantas Stanys, Ingrida Mažeikienė, Sonata Trumbeckaite, Rasa Baniene, Julius Liobikas

**Affiliations:** 1 Institute of Horticulture, Lithuanian Research Centre for Agriculture and Forestry, 54333 Babtai, Lithuania; Vidmantas.stanys@lammc.lt (V.S.); ingrida.mazeikiene@lammc.lt (I.M.); 2 Laboratory of Biochemistry, Neuroscience Institute, Lithuanian University of Health Sciences, 44307 Kaunas, Lithuania; sonata.trumbeclaite@lsmuni.lt (S.T.); rasa.baniene@lsmuni.lt (R.B.); 3 Department of Pharmacognosy, Medical Academy, Lithuanian University of Health Sciences, 44307 Kaunas, Lithuania; 4 Department of Biochemistry, Medical Academy, Lithuanian University of Health Sciences, 44307 Kaunas, Lithuania

**Keywords:** anthocyanin metabolites, antioxidants, cardioprotection, hepatoprotection, nephroprotection, neuroprotection

## Abstract

Anthocyanins are biologically active water-soluble plant pigments that are responsible for blue, purple, and red colors in various plant parts—especially in fruits and blooms. Anthocyanins have attracted attention as natural food colorants to be used in yogurts, juices, marmalades, and bakery products. Numerous studies have also indicated the beneficial health effects of anthocyanins and their metabolites on human or animal organisms, including free-radical scavenging and antioxidant activity. Thus, our aim was to review the current knowledge about anthocyanin occurrence in plants, their stability during processing, and also the bioavailability and protective effects related to the antioxidant activity of anthocyanins in human and animal brains, hearts, livers, and kidneys.

## 1. Introduction

Anthocyanins are pigments belonging to the flavonoid group, which is widely distributed in plants. They are responsible for blue, purple, and red colors in flowers, fruits, and vegetables and protect plants from environmental stresses such as high sunlight irradiance [1] or low nitrogen [2]. Chemically, anthocyanins are produced when anthocyanidins are glycosylated. The most abundant anthocyanidin in plants is cyanidin. Other anthocyanidins are less abundant, and their frequency decreases in this order: delphinidin, peonidin, pelargonidin, petunidin, and malvidin [3]. Anthocyanidins are flavylium ion derivatives that vary in terms of their substituent groups: –H, –OH, or –OCH_3_. Usually, anthocyanidins are glycosylated at the C3 or C3 and C5 sites, but the glycosylation of other sites has also been reported [4]. The biological activity of anthocyanins depends on their structure; however, all samples, including those with different compositions and amounts of anthocyanins, extracted from various berries and vegetables, are biologically active [5]. Azevedo et al. [6] established that the radical scavenging activity and reducing properties of anthocyanins strongly depend on the chemical structures of particular anthocyanins; this effect increases with the presence of catechol and pyrogallol groups in ring B of cyanidin-3-glucosides and the respective aglycones. Some studies have shown that delphinidin has the highest antioxidant activity compared with the other five anthocyanidins due to the three hydroxyl groups on the B-ring [5,7]. An increasing body of evidence shows that anthocyanin intake can have a protective effect on human and animal brains, hearts, livers, and kidneys, and many of the therapeutic effects may be purported to the antioxidant activities of anthocyanins and their metabolites [8,9,10,11,12]. The antioxidant activity of these compounds manifests through direct and indirect methods of action. Thus, anthocyanins can directly scavenge reactive oxygen species (ROS) [13,14], whereas the indirect pathways involve stimulation of the synthesis or activity of antioxidant enzymes (catalase, superoxide dismutase (SOD), glutathione peroxidase) [15]; inhibition of ROS-forming enzymes, such as nicotinamide adenine dinucleotide phosphate (NADPH) oxidase and others [16,17]; or even mild uncoupling of mitochondrial respiration preventing ROS generation [9,18]. It can also be assumed that for effective therapeutic action of anthocyanins, both the ROS scavenging activity and the modulation of cellular antioxidant systems are required [14]. Here, we review the current knowledge about anthocyanin occurrence in plants, their stability during processing, and the health benefits to humans and animals.

## 2. Natural Sources of Anthocyanins

Anthocyanins are natural, water-soluble plant pigments that are responsible for blue, red, or purple colors in plants. Plant genotypes, agro-climatic conditions, and fruit or vegetable maturity are significant factors in the composition and quantity of anthocyanins [19]. Therefore, the main sources of anthocyanins in human diet are fruits and vegetables, which accumulate anthocyanins in both the peel and flesh; however, their content varies greatly (Table 1).

Fruits, especially dark blue berries, accumulate a large total amount of anthocyanins, while vegetables tend to have lower anthocyanin concentrations. One of the highest anthocyanin concentrations was identified in bilberries, with delphinidins being the dominant anthocyanins making up over 57.6% of the total, with cyanidins representing 23.7% and malvidins representing 14.1% of the total [20]. Berries of the golden currant cultivar ‘Corona’ are the richest in anthocyanins among *Ribes* spp., with cyanidins being the dominant type [21]. Blackcurrants have a higher proportion of delphinidins, ranging from 66.7% to 70.2%, as shown in various studies [20,21]. In contrast, cyanidins dominate in redcurrant, elderberry, sweet cherry, and sour cherry, and sometimes, they are the only anthocyanins [11,20,22,23] (Mikulic-Petkovsek et al., 2014; Veberic et al., 2015; Bendokas et al., 2017, Blackhall et al., 2018). Malvidins are the main anthocyanin in grapes with a relative content ranging from 35.8% to 67.1% [25].

In terms of vegetables, red chicory has been shown to have the highest concentration of anthocyanins; however, it is 2–20 times lower than that in berries. Cyanidins are major anthocyanins in most vegetables; however, eggplant only accumulates delphinidins.

The consumption of anthocyanins varies from 9 mg/day on average in the United States to 19 mg/day in Europe, but daily consumption may reach 28 mg in some European countries [33]. The main sources of anthocyanins are berries (39% in the US and 43% in Europe), wine (18% in the US and 22% in Europe), and fruits (9% in the US and 19% in Europe), with other sources being vegetables and other foods [34].

## 3. Stability of Anthocyanins in Foods and Beverages

Anthocyanins are natural plant compounds that are increasingly being used in the food and pharmaceutical industry due to their effects on human health. However, the low stability of anthocyanins is still an obstacle to their use [35]. The stability of anthocyanins depends on the pH, temperature, light, presence of solvents and oxygen, and other factors [36]. Anthocyanins are more stable under acidic conditions. At a pH of 1.0, flavylium cations are the predominant species and contribute to the development of purple and red pigments, while at pH 2.0–4.0, the blue quinoidal species predominates. When the pH value reaches 7.0, anthocyanins are usually degraded [36]. The storage temperature affects the concentration of anthocyanins in extracts; for instance, 11% of rosella anthocyanins were lost after storage for 60 days at 4 °C, while 99% of anthocyanins were degraded in the same extracts stored at 37 °C for the same period [37].

The stability of anthocyanins from various *Ribes* species was reported to depend on their composition and storage conditions [24]. Anthocyanins in redcurrant berry extract have been shown to be more stable at room temperature and in the presence of light than extracts from berries of golden currant and gooseberry. After the storage of anthocyanin extracts under dark and cold conditions (+4 °C) for 84 days, up to 90% of redcurrant, 80% of gooseberry and golden currant, and up to 50% of blackcurrant anthocyanins remained intact [24].

Thermal food processing negatively affects the nutritional value of anthocyanin-rich juices as it results in anthocyanin degradation [38]. Thermal processing is responsible for the loss of up to 35% of anthocyanins. Even short-term thermal treatment (5 s at 85 °C) resulted in a loss of 9% of anthocyanins from strawberry juice, while pasteurization for 15 min resulted in a loss of 21% [39]. Boiling of red cabbage resulted in a loss of 41.2% of anthocyanins, while the anthocyanin concentration remained the same after steaming or stir-frying. Possibly, as highly water-soluble pigments, anthocyanins may be lost by leaching in the case of boiling [30].

Various anthocyanin stabilization methods are being developed. For instance, the improvement of anthocyanin thermal stability by yeast mannoproteins at pH 7.0 has been studied. The complexes were found to effectively protect anthocyanins from degradation during heating at 80 and 126 °C [40]. Mixing of clarified acerola juice with montmorillonite resulted in 50% more anthocyanins, regardless of time or pH changes [41]. Copigmentation is another natural tool that can be used to enhance anthocyanin stability. The most studied copigments are phenolic acids such as hydroxycinnamic and hydroxybenzoic acids [42]. Babaloo and Jamei [43] established that caffeic acid provides more stability for anthocyanins than benzoic, tannic, and coumaric acids. Encapsulation with polysaccharides, such as β-cyclodextrin, maltodextrin, or Arabic gum, is also important for the stabilization of anthocyanins. The protective effect of β-cyclodextrin was evident for all blackberry anthocyanins after thermal treatment at 90 °C for 2 h [44].

Novel techniques for phenolic compound isolation from natural products that avoid the degradation of these compounds have been developed. Block freeze concentration has been employed to extract anthocyanins from strawberries and enrich yogurt with the obtained concentrated strawberry juice. As a result, yogurt with a high anthocyanin content and greater antioxidant activity was produced; however, it had a short shelf life [45]. The foam mat drying technique is now considered to be an effective dehydration method to produce powder from juices or pulp. Only a small reduction in anthocyanins (7–9%) was observed after the storage of jambolana juice powder produced by foam mat drying for 150 days [46]. When foam-mat freeze-drying was used for powder production from blueberry juice, 80–100% of anthocyanins remained after processing; the most stable was Cy3glc [47].

## 4. Bioavailability of Anthocyanins

The beneficial health effects of anthocyanins strongly depend on their bioavailability, as only a small fraction of anthocyanins is absorbed by human body [48,49]. The concentration of anthocyanins in the plasma reaches a maximum value within 0.5–2 h after the consumption of anthocyanin-rich foods [50]. A low concentration of anthocyanins in plasma and urine has been observed in several studies. Less than 2% of original anthocyanins may be found in the plasma or urine after consumption; however, anthocyanins go through several transformations in the small and large intestines; thus, only a small fraction of the anthocyanins remains nonmetabolized or catabolized [51]. Mueller et al. [52] stated that a complex mixture of anthocyanin metabolites in the plasma rather than a single type of anthocyanins may cause beneficial effects in humans.

Gamel et al. [32] evaluated the absorption of anthocyanin metabolites after the consumption of purple wheat crackers and bars. They established that the total concentration of anthocyanin metabolites in urine peaked at 0–2 and 2–4 h, with 18–22 ng/mL excreted on average. For both products, the total amount of accumulated anthocyanins reached 13 µg in 24 h, representing 0.19%. A significant difference between male and female participants was also observed: males excreted 17.1 μg, while females excreted 11.6 μg of anthocyanin metabolites in a 24-h period. Krga and Milenkovic [53] conducted a comprehensive analysis of 20 studies on the anthocyanin concentration in human plasma after the ingestion of food, extracts, and drinks. They found that anthocyanin adsorption was the fastest after drinking red wine and red grape juice. The maximum concentration in the plasma was reached in 0.3 and 0.5 h, respectively, while the maximum concentration of anthocyanins from blueberry powder was reached in 4 h. Interestingly, the maximum anthocyanin concentration in plasma varied from 1.4 to 591.7 nM and did not depend on the administered dose, which was possibly due to differences in the anthocyanin composition in various foods. Recently, a human pilot study was performed by Rȯhrig and colleagues [54] on five healthy male volunteers who were consuming anthocyanin-rich blackcurrant extract. They studied the kinetics of the dominant anthocyanins—Dp3rut and Cy3rut—in plasma and urine after the consumption of blackcurrant extract. The peak concentrations of both anthocyanins in plasma were reached within 2 h after consumption. The maximum concentrations were 8.6 ± 5.8 nmol/L for Dp3rut and 9.8 ± 3.1 nmol/L for Cy3rut, which later gradually decreased. Similarly, urinary excretion rates of both studied anthocyanins peaked within 0−2 h of ingestion and reached 20.0 ± 2.6 nmol/h (Dp3rut) and 21.2 ± 3.8 nmol/h (Cy3rut). The total excreted amount was calculated: 0.040% (Dp3rut) and 0.048% (Cy3rut) of the ingested doses. In addition, after consumption of the anthocyanin-rich extract, the concentration of the main metabolite, protocatechuic acid, increased significantly [54].

In humans, anthocyanins consumed with food may be digested in the gastrointestinal tract, absorbed into the blood, and later metabolized [55]. Some studies have stated that minute amounts of anthocyanins are absorbed in the small intestine. The majority of ingested anthocyanins reach the large intestine where they are metabolized [56] and where structural modifications (deglycolysation, degradation, hydroxylation, etc.) take place due to the changing physiological conditions. Anthocyanins can also be further modified by various enzymes in the small intestine before entering the bloodstream [57]. Twenty-two metabolites of Cy3glc were identified using an isotopic approach [58]. Among them, phloroglucinaldehyde, 3,4-dihydroxybenzaldehyde, and hydroxybenzoic acid were found to be produced as phase I metabolites, while the phase II metabolites identified were mainly glucuronidated and methylated cyanidins. Colonic anthocyanin metabolites included hydroxybenzoic acid, hippuric acid, phenylpropenoic acid, ferulic acid, and phase II protocatechuic acid conjugates [58,59,60]. Bresciani and colleagues [61] analyzed the catabolism of anthocyanin-rich elderberry extract with different gut microbial strains in vitro and established that their metabolic pathways were different. Some common metabolites were found among all studied strains; however, each of them had several phenolic metabolites produced specifically by that strain. These in vitro results could provide new knowledge of the variability in anthocyanin metabolism in different organisms. After absorption, the fast transport of anthocyanins and their metabolites to the liver, heart, lungs, brain, and kidneys may be observed [55,62].

Sandoval-Ramírez and colleagues [63] summarized studies on the anthocyanin concentration in various animal tissues and stated that in short-term experiments, after a single dose of bilberry extract, the highest concentration of cyanidin-3-*O*-glucoside (40.46 pmol/g) was found in the brain after 0.25 min [62]. In addition, Cy3glc was identified in other tissues, such as the gastrointestinal tract (8 × 105 pmol/g), lungs (5.19 × 104 pmol/g), and prostate (4 × 104 pmol/g), with peak concentrations occurring at different times after a single oral dose of the extract [63,64]. In long-term studies, the highest concentration of Mv3glc (4.43 pmol/g) was established in the brains of pigs, while the concentration of Pt3glc reached 6.66 pmol/g [63]. Cy3ara from tart cherry was identified in the hearts, brains, livers, kidneys, and bladders of Wistar rats; however, its concentration was low and ranged from 2.28 × 10^−4^ to 1.16 × 10^−3^ pmol/g [65]. In general, Cy3glcand its metabolites are the most abundant anthocyanins in animal tissues, and Cy3glcmay be one of the most promising bioactive molecules for human health [63]. It is also worth noting that care is needed to avoid artefacts when studying and evaluating antioxidant effects of bioavailable anthocyanins in model organisms and the human body, as findings can depend on the chosen marker, the sensitivity of method to detect ROS and to measure oxidative damage, or even on the changes in plasma urate concentrations [66]). It is not the aim of the present review to analyze the suitability of methods to evaluate the antioxidant activity of anthocyanins; thus, for the complexity of the matter, one could consult several excellent reviews [66,67,68,69,70].

## 5. Biological Effects Related to the Antioxidant Activity of Anthocyanins—In Vivo Studies in Model Organisms

### 5.1. Neuroprotection

At the animal level, oral administration of anthocyanin-rich berry extract of *Vaccinium myrtillis* L. (100 mg/kg for 7 days) has been shown to suppress psychological stress-induced cerebral oxidative stress and dopamine abnormalities in distressed mice [71]. Anthocyanins extracted from black soybeans have also been demonstrated to reverse D-galactose- or lipopolysaccharide (LPS)-induced oxidative stress, neuroinflammation, and neurodegeneration in adult murine models [72,73,74]. Likewise, the same anthocyanins were shown to reduce elevation of the ROS level and consequent oxidative stress induced by the amyloid beta oligomer (Aβ 1-42) through stimulating the intracellular antioxidant system (the transcription factor E2-related factor 2 (Nrf2) and heme oxygenase-1 (HO-1) pathways). These anthocyanins also prevented apoptosis and neurodegeneration by suppressing the apoptotic and neurodegenerative markers in the amyloid precursor protein/presenilin-1 (APP/PS1) mouse model of Alzheimer’s disease (AD) [75].

Positive findings have also been reported by Qin et al. [76] in Cy3glc-treated (10 mg/kg for 30 days) rats injected with Aβ 1-42, which showed an attenuation of Aβ- and oxidative stress-induced GSK-3β hyperactivation and hyperphosphorylation of the tau protein. These findings are in agreement with a study in which rats were injected with Aβ 1-42 bilaterally into the hippocampal CA1 area in order to produce an animal model of AD [77]. It was observed that the memory impairment was reduced in rats that received 80 mg/kg of *Lycium ruthenicum* Murr. anthocyanin-rich extract in comparison to other AD model rats. Moreover, the anthocyanin extract enhanced the activities of total SOD and catalase and increased the glutathione concentration in serum and brain tissues.

Chen et al. [78] also confirmed that *Lycium ruthenicum* Murr. anthocyanins can exert neuroprotective effects in D-galactose (D-gal)-treated rats. Anthocyanins reduce the level of receptor for advanced glycation end products (RAGE), suppress oxidative stress, and reduce levels of inflammation markers such as nuclear factor kappa B (NF-κB), interleukin-1-β (IL-1β), cyclooxygenase-2 (COX-2), and tumor necrosis factor-α (TNF-α), among others. Sustained levels of antioxidant enzymes (SOD and catalase), a decreased concentration of the lipid peroxidation product malondialdehyde, and significantly decreased expression of aging-associated monoamine oxidase-B in D-gal-treated mice were also demonstrated after the administration (30 mg/kg and 60 mg/kg) of black rice anthocyanins [79]. Anthocyanins from fruits of *Aronia melanocarpa* (Michx.) Elliot (30 mg/kg) retained the levels of total SOD and glutathione peroxidase and inhibited the excessive accumulation of inflammatory cytokines in the brain tissue of D-gal-treated mice [80]. Furthermore, recently, a study using a model of streptozotocin-induced dementia of sporadic AD [81] proposed that pretreatment with a commercial anthocyanin extract from grape skins (200 mg/kg for 25 days) can prevent behavioral alterations and protect against the changes in ROS and antioxidant enzyme (SOD, catalase, and glutathione peroxidase) levels.

Notably, purified anthocyanins and anthocyanidins have also been found to exert neuroprotective activities. For instance, pre-treatment with pelargonidin (Pg) (20 mg/kg) significantly suppressed the formation of thiobarbituric acid reactive substances, indicating reduced lipid peroxidation in 6-hydroxydopamine-lesioned rats (an experimental model or Parkinson’s disease) [82]. Furthermore, orally consumed Cy3glc (2 mg/kg) reduced brain superoxide levels, infarct size, and improved neurological functions, thus revealing a neuroprotective effect in the cerebral artery occlusion model of ischemia in mice [83]. A recent report [84] also showed that an intravenous injection of Cy3gal and Cy3glc (0.025 mg/kg and 0.05 mg/kg), but not Cy3rut, in rats protected against ischemia-induced caspase-3 activation and necrotic cell death, as well as reducing the infarct size in the cerebral cortex and cerebellum. In contrast, 0.025 mg/kg of Pg3glc had no effect of the activity of caspase-3 but reduced the infarct size. The effects of anthocyanins have been found to correlate with the cytochrome c reducing capacity, and Pg3glc has been shown to have the smallest effect among tested anthocyanins. Thus, authors have proposed that under certain conditions, such as ischemic brain damage, the reducing properties rather than antioxidant properties of anthocyanins might be important in providing neuroprotection.

### 5.2. Cardioprotection

Since it is known that bilberries and blackcurrant berries are rich in anthocyanins, Brader et al. [85] investigated the effects of a berry-enriched diet (5 g of berry powder containing 172 mg of anthocyanins per day) on lipid profiles and other biomarkers in Zucker diabetic fatty rats. The results after eight weeks of supplementation demonstrated reduced levels of total and LDL-cholesterol, which was partly due to the altered expression of hepatic liver X receptor-α. Using an in vivo model of coronary occlusion and reperfusion, another study showed that the infarct size was reduced in the hearts of rats that received a long-term purple maize anthocyanin-enriched diet (with Cy and Pg glycosides as the main components) [86]. The authors proposed that the observed cardioprotection could be associated with increased myocardial glutathione levels and thus an improved endogenous cardiac antioxidant defense system. Moreover, Ziberna et al. [87] demonstrated that bilberry anthocyanins could exert concentration-dependent responses on whole rat hearts under ischemia–reperfusion (I-R) conditions. Thus, at low concentrations (0.01–1 mg/L, expressed as Cy3glc equivalents), the extent of I-R injury was significantly reduced, whereas at 5–50 mg/L, anthocyanins showed cardiotoxic activity despite having an intracellular antioxidant capability that increased in a concentration-dependent manner.

In addition, recent evidence from an LPS-induced myocardial injury model in mice [88] showed that pure anthocyanins such as Cy3glc could restore the activity of the mitochondrial electron transport chain (namely, the activity of complexes I and II) and thus significantly attenuate ROS production. Furthermore, the anthocyanins ameliorated cardiac injury, cell death, and improved cardiac function. It is worth noting that Cy3glc also suppressed the expression of endotoxin-induced pro-inflammatory cytokines and the level of protein nitration while elevating the intracellular level of reduced glutathione.

In general, these observations suggest that the cardioprotective activities of anthocyanins may not be solely attributed to their antioxidant properties; therefore, a broader view should be implemented [18,87,89,90].

### 5.3. Hepatoprotection

The liver plays an important role in the metabolic elimination of drugs and toxic compounds known to cause injury and reduce the function of the liver. Arjinajarn et al. [91] observed that rice bran extract (250–1000 mg/kg) rich in Cy3glc and Pn3glc (13.24 and 5.33 mg/g of crude extract, respectively) significantly prevented gentamicin-induced intoxication of the liver. It has been shown that an anthocyanin-rich extract significantly reduced the hepatic malondialdehyde level (a biomarker for oxidized lipids), increased expression of the antioxidant enzyme SOD, and prevented elevation of levels of liver injury markers, namely alanine and aspartate aminotransferase. These effects were related to the suppression of both the oxidative pathway regulated by the transcription factor Nrf2 and the inflammatory pathway regulated by NF-κB. The authors proposed that Cy3glc, the major anthocyanin in this extract, is responsible for these antioxidant and anti-inflammatory properties [91]. Moreover, Hou et al. found that black rice bran extract, which is rich in anthocyanins (mainly Cy3glc and Pn3glc), protected mice liver intoxicated with carbon tetrachloride (CCl_4_) [87]. It was found that mice treated with black rice extract (200–800 mg/kg) showed increased SOD and glutathione peroxidase activities and increased levels of reduced glutathione as compared with a CCl_4_-intoxicated model group [92]. Similar results about the protective effects of blueberry anthocyanin extract (anthocyanin content up to 25%) on CC1_4_-induced liver injury in mice in vivo were presented by [93]. The extract had reduced concentrations of alanine aminotransferase, aspartate aminotransferase, and malondialdehyde in a dose-dependent manner, while the activities of SOD, catalase, and glutathione reductase increased [93]. Blackberry fruit extract was similarly shown to attenuate lipid peroxidation and to recover the activity of antioxidant enzymes in CC1_4_-treated rats [94]. Recently, Sun et al. observed that anthocyanins from blueberry (100 mg/kg and 200 mg/kg per day) protected mouse liver from CCl_4_-induced hepatic fibrosis [95]. It was detected that blueberry anthocyanins reduced ROS generation and tissue oxidative damage, decreased inflammation, and suppressed the activity of hepatic stellate cells. Interestingly, the activity of mitochondrial electron transport chain complexes I and II was also restored after treatment with anthocyanins [95].

It is also known that liver inflammation and an excessive accumulation of lipids play critical roles in the pathogenesis of alcoholic liver diseases. Accordingly, in a recent study, Cy3glc extracted from *Lonicera caerulea* L. was shown to exert a hepatoprotective effect on alcoholic steatohepatitis in mice [96]. Zuo et al. detected substantial decreases in serum aminotransferases and triglycerides and found increased albumin levels after treatment. In addition, anthocyanidins significantly suppressed the expression of SREBP1 (a transcription factor involved in lipogenesis) and enhanced the phosphorylation of AMPK as compared with chronic ethanol administration. Cy-3-g suppressed inflammasome activation, thereby preventing activated macrophages from producing pro-inflammation cytokines [96]. Moreover, another study demonstrated that a phenolic fraction of *Lonicera caerulea* L. ameliorated inflammation and lipid peroxidation by upregulating Nrf2 and SOD and downregulating the transcription factor forkhead box protein O1 and HO-1 in a mouse model of nonalcoholic steatohepatitis (NASH) induced by a high-fat diet in combination with CCl_4_ [97]. Prokop et al. also showed in vivo that a blue grain bread wheat-based diet (genotype UC66049, containing 121 mg of Cy3glc per kg of wheat) increased the activity of the microsomal xenobiotic-metabolizing system cytochrome P450 by 20–50% in rats after 72 days of intake. In addition, an anthocyanin-rich diet significantly increased the antioxidant power of plasma as shown by a FRAP assay and the level of total –SH groups in plasma when compared with the control [98].

### 5.4. Nephroprotective Effects

Anthocyanins are considered to be a functional food factor and to play an important role in the prevention of kidney diseases. For example, a recent study [99] investigated the effects of anthocyanin-rich bilberry extract (200 mg/kg daily) on the antioxidant status of animals intoxicated with CCl_4_. Rats received the extract orally for 7 days, and on the last day, a single dose of CCl_4_ was intraperitoneal (i.p.) injected. It was found that pretreatment with the anthocyanin-rich extract resulted in a significant reduction in pro-oxidative (H_2_O_2_, oxidized glutathione, xanthine oxidase) and pro-inflammatory markers (myeloperoxidase, nitric oxide, and TNF-α), and a substantial increase of antioxidant enzyme levels (catalase, SOD, glutathione peroxidase, and S-transferase). Moreover, the anthocyanins significantly reduced the degree of damage to the proximal and distal tubules in the kidney cortex [99].

Positive findings have also been reported by another study [100]. It showed that the administration of *Malva sylvestris* L. extract (200 or 400 mg/kg) rich in anthocyanins reduced the renal toxicity induced by gentamicin and thus led to (i) an improvement in kidney function, (ii) a decrease in the expression levels of pro-inflammatory markers (TNF-α), (iii) a reduction in oxidative stress (levels of malondialdehyde and total antioxidant capacity), and (iv) a decrease in tissue injuries.

Similar beneficial effects of the bilberry diet (100 mg/kg daily) on the levels of serum malondialdehyde, catalase, and advanced oxidation protein products were demonstrated in a rat model of gentamicin-induced nephrotoxicity, and the effects correlated well with the antioxidant activity (assessed in vivo and in vitro) as well as with high anthocyanin levels [101]. It is worth noting that anthocyanin-rich fruits of *Panax ginseng* Meyer have also been shown to attenuate cisplatin-induced elevations in blood urea nitrogen and creatinine levels as well as the prevalence of histopathological injuries in mice [102]. The positive outcomes are related to reduced levels of malondialdehyde, HO-1, cytochrome P450 E1, 4-hydroxynonenal, TNF-α, and IL-1β, as well as concomitantly increased levels of reduced glutathione, catalase, and SOD.

In addition, Lee et al. [103] recently revealed that intravenously administered pure Pg (0.4 mg/kg) could modulate renal function in a mouse model of sepsis. Treatment with Pg reduced renal tissue injury, plasma nitrite and nitrate production, and TNF-α, IL-6, myeloperoxidase, and malondialdehyde levels. The total glutathione content as well as the activity of antioxidant enzymes such as SOD, glutathione peroxidase, and catalase in kidney tissues were also found to be restored after Pg injection.

## 6. Biological Effects Related to the Antioxidant Activity of Anthocyanins—In Vivo Studies in Humans

Epidemiological and clinical studies suggest that an anthocyanin-enriched diet may lower levels of certain oxidative stress biomarkers in humans, and this could be associated with reduced risk of cognitive decline and the development of neurodegenerative and cardiovascular diseases, as well as having sustained hepatic function and kidney protecting activities [12,104,105,106,107,108,109,110,111,112,113,114,115].

### 6.1. Antioxidant and Anti-Atherosclerogenic Effects

A randomized clinical trial [116] evaluated the effects of a standardized maqui berry (*Aristotelia chilensis* (Mol.) Stuntz) extract (containing 162 mg of anthocyanins) on products of lipid peroxidation in healthy, overweight, and smoker adults. The results suggested that supplementation with the extract can be related to a limited term (max for 40 days) reduction in oxidized low-density lipoprotein (LDL) levels and a decrease in urinary F2-isoprostanes. Another study [117] concluded that the acute consumption of anthocyanin-rich red *Vitis labrusca* L. grape juices could be related to decreased levels of thiobarbituric acid reactive substances and lipid peroxides in the serum of healthy subjects. It has also been demonstrated that regular (for 30 days) anthocyanin-rich sour cherry consumption could suppress the formation of ROS by circulating phagocytes and decrease the risk of systemic imbalance between oxidants and antioxidants [118]. It is worth noting that a portion (300 g) of blueberries, the dietary source of anthocyanins provided to young volunteers involved in a randomized cross-over study, significantly reduced H_2_O_2_-induced DNA damage in blood mononuclear cells [119]. In another human pilot intervention study, the consumption of anthocyanin-rich bilberry (*Vaccinium myrtillius* L.) pomace extract was found to modulate transcription factor E2-related factor 2 (Nrf2)-dependent gene expression in peripheral blood mononuclear cells [120].

A single-blind randomized placebo-controlled intervention trial, which lasted for 8 weeks and involved 72 unmedicated subjects, revealed that the administration of various berries (including bilberries, chokeberries, and blackcurrants) increased both the concentration of high-density lipoprotein (HDL) cholesterol and the plasma antioxidant capacity [121]. Higher dietary anthocyanin and flavan-3-ol intake was associated with anti-inflammatory effects in 2375 Framingham Heart Study Offspring Cohort participants [122]. Interestingly, the consumption of 300 mL of red wine (a total dose of anthocyanins was 304 µM, which was the highest amount among detected compounds) with a meal was shown to prevent the postprandial increases in plasma lipid hydroperoxides and cholesterol oxidation products and therefore protect against a potential pro-atherosclerogenic effect [123]. Similar findings were obtained in a randomized cross-over trial, which concluded that a moderate consumption of red wine decreases erythrocyte SOD activity [124]. In another randomized double-blind trial, 150 subjects with hypercholesterolemia consumed a purified anthocyanin mixture derived from bilberries and blackcurrants (320 mg/day) for 24 weeks [125]. It was found that anthocyanin consumption significantly decreased the levels of inflammatory biomarkers (C-reactive protein, soluble vascular cell adhesion molecule-1, and plasma IL-1β) and increased the HDL cholesterol level. Recently, it was shown that a daily intake of 150 g of anthocyanin-rich blueberries resulted in clinically relevant improvements in endothelial function and systemic arterial stiffness, which was probably due to the improved nitric oxide bioactivity and HDL status [126].

### 6.2. Hepatoprotective Benefits

Nonalcoholic fatty liver disease (NAFLD), defined by excessive lipid accumulation in the liver, is the hepatic manifestation of insulin resistance and metabolic syndrome. NAFLD encompasses a wide spectrum of liver diseases ranging from simple uncomplicated steatosis to steatohepatitis, cirrhosis, and hepatocellular carcinoma [106]. Zhang et al. [127] reported that anthocyanins extracted from bilberry and blackcurrant (320 mg/day) and administered for 12 weeks ameliorated liver injury in patients with NAFLD. It was observed that a so-called “anthocyanin group” exhibited significant decreases in the plasma alanine aminotransferase, cytokeratin-18 M30 fragment, and myeloperoxidase levels. It was also found that consumption of *Myrica rubra* Sieb. and Zucc. juice (250 mL for 4 weeks) protected young adults (18–25 years old) against NAFLD by improving the plasma antioxidant status and inhibiting the inflammatory and apoptotic responses involved in this disease [128].

### 6.3. Nephroprotection

In another study, red fruit juice (40% red grape juice, 20% blackberry juice, 15% sour cherry juice, 15% blackcurrant juice, and 10% elderberry juice) with high polyphenol and anthocyanin contents was tested for its preventive potential in hemodialysis patients [129]. For this purpose, 21 subjects consumed 200 mL/day of juice according to the following protocols: 3-week run-in; 4-week juice uptake; 3-week wash-out. The results revealed a significant decrease in DNA oxidation damage and protein and lipid peroxidation and an increase in the reduced glutathione level; the effects were attributed to the high anthocyanin and polyphenol contents of the juice [129]. Another study [130] demonstrated that the regular consumption of concentrated red grape juice by hemodialysis patients could be associated with the reduced neutrophil NADPH oxidase activity and plasma concentrations of oxidized LDL and inflammatory biomarkers.

## 7. Concluding Remarks

In conclusion, anthocyanins are valuable biomolecules with a broad variety of biological effects on human health, and we suggest adding more anthocyanin rich fruits, vegetables, and their products to the daily diet. Numerous studies indicate that bilberry, blackcurrant, elderberry, and other berries have the highest total concentrations of anthocyanins; therefore, the consumption of fresh berries and their processed products may have greater beneficial effects on humans. Various anthocyanin stabilization methods have been developed e.g., copigmentation with other phenolic acids, encapsulation with polysaccharides, block freeze concentration, and powder production using the foam mat drying technique. All of them enable anthocyanins to be preserved during processing, thus increasing their bioavailability and delivery to target tissues in the human body. However, long-term studies on the impacts of anthocyanin-rich product consumption on human health are still rare. Further studies could focus on the identification and tracking of individual anthocyanin metabolites and on the determination of the exact dosage and delivery platforms sustaining the antioxidant properties of anthocyanins in vivo. In addition, as an alternative to natural sources, synthesis of the most bioactive anthocyanins in bioreactors should be considered.

## Figures and Tables

**Table 1:** Maximum amount of anthocyanins (mg 100 g^−1^ of fresh weight (FW)) in fruits and vegetables.

Source	Anthocyanin Amount mg 100 g^−1^ FW	Dominant Anthocyanins	References
Bilberry	772.4	Dp3gal, Dp3glc, Dp3ara, Mv3glc, Cy3gal, Cy3glc, Cy3ara	[20]
Blackcurrant	478.6	Dp3rut, Cy3rut, Dp3glc, Cy3glc	[21,22]
Golden currant	615.5	Cy3rut, Cy3glc, Pn3rut	
Redcurrant	66.7	Cy3glc, Cy3rut, Cy3sam	
Elderberry	580.0	Cy3sam, Cy3glc	[19]
Grapes	116.4	Mv3glc, Cy3glc, Dp3glc, Pt3glc, Pn3glc	[23]
Sour cherry	147.0	Cy3rut	[24]
Sweet cherry	244.0	Cy3rut, Pn3rut	[25]
Wild strawberry	10.0	Pg3glc, Cy3glc	[26]
Black carrot	126.4	Cy3xylglcgal, Cy3xylgal	[27]
Eggplant	8.7	Dp3glc, Dp3rut,	[28,29]
Red cabbage	23.4	Cy3glc, Cy3rut, Dp3glc, Dp3rut, Cy3diglc5glc	[30,31]
Red chicory	39.2	Cy3glc	[29]
Purple wheat	23.5	Cy3glc	[32]

Cy—cyanidin; Dp—delphinidin; Mv—malvidin; Pg—pelargonidin; Pn—peonidin; Pt—petunidin; ara—arabinoside; gal—galactoside; glc—glucoside; rut—rutinoside; sam—sambubioside; xyl—xyloside.

## References

[1] Landi M., Tattini M., Gould K.S. (2015). Multiple functional roles of anthocyanins in plant-environment interactions. Environ. Exp. Bot..

[2] Liang J., He J. (2018). Protective role of anthocyanins in plants under low nitrogen stress. Biochem. Biophys. Res. Commun..

[3] Cody R.B., Tamura J., Downard K.M. (2018). Quantitation of anthocyanins in elderberry fruit extracts and nutraceutical formulations with paper spray ionization mass spectrometry. J. Mass Spectrom..

[4] Zhao C.L., Chen Z.J., Bai X.S., Ding C., Long T.J., Wei F.G., Miao K.R. (2014). Structure–activity relationships of anthocyanidin glycosylation. Mol. Divers..

[5] Blando F., Calabriso N., Berland H., Maiorano G., Gerardi C., Carluccio M.A., Andersen Ø.M. (2018). Radical Scavenging and Anti-Inflammatory Activities of Representative Anthocyanin Groupings from Pigment-Rich Fruits and Vegetables. Int. J. Mol. Sci..

[6] Azevedo J., Fernandes I., Faria A., Oliveira J., Fernandes A., de Freitas V., Mateus N. (2010). Antioxidant properties of anthocyanidins, anthocyanidins 3-glucosides and respective portisins. Food Chem..

[7] Ali M.H., Almagribi W., Al-Rashidi M.N. (2016). Antiradical and reductant activities of anthocyanidins and anthocyanins, structure-activity relationship and synthesis. Food Chem..

[8] Pojer E., Mattivi F., Johnson D., Stockley C.S. (2013). The Case for Anthocyanin Consumption to Promote Human Health: A Review. Compr. Rev. Food Sci. Food Saf..

[9] Liobikas J., Skemiene K., Trumbeckaite S., Borutaite V. (2016). Anthocyanins in Cardioprotection: A Path through Mitochondria. Pharmacol. Res..

[10] Kalt W. (2019). Anthocyanins and Their C_6_-C_3_-C_6_ Metabolites in Humans and Animals. Molecules.

[11] Bendokas V., Skemiene K., Trumbeckaite S., Stanys V., Passamonti S., Borutaite V., Liobikas J. (2019). Anthocyanins: From Plant Pigments to Health Benefits at Mitochondrial Level. Crit. Rev. Food Sci. Nutr..

[12] Kalt W., Cassidy A., Howard L.R., Krikorian R., Stull A.J., Tremblay F., Zamora-Ros R. (2020). Recent Research on the Health Benefits of Blueberries and Their Anthocyanins. Adv. Nutr..

[13] Miguel M.G. (2011). Anthocyanins: Antioxidant and/or anti-inflammatory activities. J. Appl. Pharm. Sci..

[14] Ereminas G., Majiene D., Sidlauskas K., Jakstas V., Ivanauskas L., Vaitiekaitis G., Liobikas J. (2017). Neuroprotective Properties of Anthocyanidin Glycosides Against H_2_O_2_-induced Glial Cell Death Are Modulated by Their Different Stability and Antioxidant Activity In Vitro. Biomed. Pharmacother..

[15] Aboonabi A., Singh I. (2015). Chemopreventive Role of Anthocyanins in Atherosclerosis via Activation of Nrf2-ARE as an Indicator and Modulator of Redox. Biomed. Pharmacother..

[16] Lim T.G., Jung S.K., Kim J., Kim Y., Lee H.J., Jang T.S., Lee K.W. (2013). NADPH Oxidase Is a Novel Target of Delphinidin for the Inhibition of UVB-induced MMP-1 Expression in Human Dermal Fibroblasts. Exp. Dermatol..

[17] Reis J.F., Monteiro V.V.S., de Souza Gomes R., do Carmo M.M., da Costa G.V., Ribera P.C., Monteiro M.C. (2016). Action Mechanism and Cardiovascular Effect of Anthocyanins: A Systematic Review of Animal and Human Studies. J. Transl. Med..

[18] Skemiene K., Rakauskaite G., Trumbeckaite S., Liobikas J., Brown G.C., Borutaite V. (2013). Anthocyanins block ischemia-induced apoptosis in the perfused heart and support mitochondrial respiration potentially by reducing cytosolic cytochrome c. Int. J. Biochem. Cell Biol..

[19] Mikulic-Petkovsek M., Schmitzer V., Slatnar A., Todorovic B., Veberic R., Stampar F., Ivancic A. (2014). Investigation of anthocyanin profile of four elderberry species and interspecific hybrids. J. Agric. Food Chem..

[20] Veberic R., Slatnar A., Bizjak J., Stampar F., Mikulic-Petkovsek M. (2015). Anthocyanin composition of different wild and cultivated berry species. LWT.

[21] Stanys V., Bendokas V., Rugienius R., Sasnauskas A., Frercks B., Mažeikienė I., Šikšnianas T. (2019). Management of anthocyanin amount and composition in genus *Ribes* using interspecific hybridisation. Sci. Hortic..

[22] Siksnianas T., Bendokas V., Rugienius R., Sasnauskas A., Stepulaitiene I., Stanys V. (2013). Anthocyanin content and stability in *Ribes* species and interspecific hybrids. Rural Dev..

[23] Dimitrovska M., Bocevska M., Dimitrovski D., Murkovic M. (2011). Anthocyanin composition of Vranec, Cabernet Sauvignon, Merlot and Pinot Noir grapes as indicator of their varietal differentiation. Eur. Food Res. Technol..

[24] Bendokas V., Stepulaitiene I., Stanys V., Siksnianas T., Anisimoviene N. (2017). Content of anthocyanin and other phenolic compounds in cherry species and interspecific hybrids. Acta Hortic..

[25] Blackhall M.L., Berry R., Davies N.W., Wallsa J.T. (2018). Optimized extraction of anthocyanins from Reid Fruits’ *Prunus avium* ‘Lapins’ cherries. Food Chem..

[26] Rugienius R., Bendokas V., Kazlauskaitė E., Siksnianas T., Stanys V., Kazanaviciute V., Sasnauskas A. (2016). Anthocyanin content in cultivated Fragaria vesca berries under high temperature and water deficit stress. Acta Hortic..

[27] Algarra M., Fernandes A., Mateus N., de Freitas V., da Silva J.C.G., Casado E.J. (2014). Anthocyanin profile and antioxidant capacity of black carrots (*Daucus carota* L. ssp. *sativus* var. *atrorubens* Alef.) from Cuevas Bajas, Spain. J. Food Compost. Anal..

[28] Liu Y., Tikunov Y., Schouten R.E., Marcelis L.F.M., Visser R.G.F., Bovy A. (2018). Anthocyanin Biosynthesis and Degradation Mechanisms in Solanaceous Vegetables: A Review. Front. Chem..

[29] Frond A.D., Iuhas C.I., Stirbu I., Leopold L., Socaci S., Andreea S., Ayvaz H., Andreea S., Mihai S., Diaconeasa Z. (2019). Phytochemical Characterization of Five Edible Purple-Reddish Vegetables: Anthocyanins, Flavonoids, and Phenolic Acid Derivatives. Molecules.

[30] Murador D.C., Mercadante A.Z., de Rosso V.V. (2016). Cooking techniques improve the levels of bioactive compounds and antioxidant activity in kale and red cabbage. Food Chem..

[31] Tong T., Niu Y.H., Yue Y., Wu S.-C., Ding H. (2017). Beneficial effects of anthocyanins from red cabbage (*Brassica oleracea* L. var. *capitata* L.) administration to prevent irinotecaninduced mucositis. J. Funct. Foods.

[32] Gamel T.H., Wright A.J., Tucker A.J., Pickard M., Rabalski I., Podgorski M., Di Ilio N., O’Brien C., Abdel-Aal E.M. (2019). Absorption and metabolites of anthocyanins and phenolic acids after consumption of purple wheat crackers and bars by healthy adults. J. Cereal Sci..

[33] Vogiatzoglou A., Mulligan A.A., Lentjes M.A.H., Luben R.N., Spencer J.P.E., Schroeter H., Khaw K.-T., Kuhnle G.G.C. (2015). Flavonoid Intake in European Adults (18 to 64 Years). PLoS ONE.

[34] Kim K., Vance T.M., Chun O.K. (2016). Estimated intake and major food sources of flavonoids among US adults: Changes between 1999–2002 and 2007–2010 in NHANES. Eur. J. Nutr..

[35] Tan C., Selig M.J., Lee M.C., Abbaspourrad A. (2018). Polyelectrolyte microcapsules built on CaCO_3_ scaffolds for the integration, encapsulation, and controlled release of copigmented anthocyanins. Food Chem..

[36] Castañeda-Ovando A., Pacheco-Hernández M.L., Páez-Hernández M.E., Rodríguez J.A., Galán-Vidal C.A. (2009). Chemical studies of anthocyanins: A review. Food Chem..

[37] Sinela A., Rawat N., Mertz C., Achir N., Fulcrand H., Dornier M. (2017). Anthocyanins degradation during storage of Hibiscus sabdariffa extract and evolution of its degradation products. Food Chem..

[38] Wang Z., Zhang M., Wu Q. (2015). Effects of temperature, pH, and sunlight exposure on the color stability of strawberry juice during processing and storage. LWT.

[39] Weber F., Larsen L.R. (2017). Influence of fruit juice processing on anthocyanin stability. Food Res. Int..

[40] Wu J., Guan Y., Zhong Q. (2015). Yeast mannoproteins improve thermal stability of anthocyanins at pH 7.0. Food Chem..

[41] Ribeiro H.L., de Oliveira A.V., de Brito E.S., Ribeiro P.R.V., Filho M.M.S., Azeredo H.M.C. (2018). Stabilizing effect of montmorillonite on acerola juice anthocyanins. Food Chem..

[42] Bimpilas A., Panagopoulou M., Tsimogiannis D., Oreopoulou V. (2016). Anthocyanin copigmentation and color of wine: The effect of naturally obtained hydroxycinnamic acids as cofactors. Food Chem..

[43] Babaloo F., Jamei R. (2018). Anthocyanin pigment stability of Cornus mas–Macrocarpa under treatment with pH and some organic acids. Food Sci. Nutr..

[44] Fernandes A., Rocha M.A.A., Santos L.M.N.B.F., Brás J., Oliveira J., Mateus N., de Freitas V. (2018). Blackberry anthocyanins: β-Cyclodextrin fortification for thermal and gastrointestinal stabilization. Food Chem..

[45] Jaster H., Arend G.D., Rezzadori K., Chaves V.C., Reginatto F.H., Petrus J.C.C. (2018). Enhancement of antioxidant activity and physicochemical properties of yogurt enriched with concentrated strawberry pulp obtained by block freeze concentration. Food Res. Int..

[46] de Carvalho Tavares I.M., Sumere B.R., Gómez-Alonso S., Gomes E., Hermosín-Gutiérrez I., Da-Silva R., Lago-Vanzela E.S. (2020). Storage stability of the phenolic compounds, color and antioxidant activity of jambolan juice powder obtained by foam mat drying. Food Res. Int..

[47] Darniadi S., Ifie I., Ho P., Murray B.S. (2019). Evaluation of total monomeric anthocyanin, total phenolic content and individual anthocyanins of foam-mat freeze-dried and spray-dried blueberry powder. J. Food Meas. Charact..

[48] Faria A., Pestana D., Azevedo J., Martel F., de Freitas V., Azevedo I., Mateus N., Calhau C. (2009). Absorption of anthocyanins through intestinal epithelial cells – putative involvement of GLUT2. Mol. Nutr. Food Res..

[49] Braga A.R.C., Murador D.C., de S Mesquita L.M., De Rosso V.V. (2018). Bioavailability of anthocyanins: Gaps in knowledge, challenges and future research. J. Food Composit. Anal..

[50] Fang J. (2014). Bioavailability of anthocyanins. Drug Metab. Rev..

[51] Eker M.E., Aaby K., Budic-Leto I., Rimac Brnčić S., El S.N., Karakaya S., Simsek S., Manach C., Wiczkowski W., de Pascual-Teresa S. (2020). A Review of Factors Affecting Anthocyanin Bioavailability: Possible Implications for the Inter-Individual Variability. Foods.

[52] Mueller D., Jung K., Winter M., Rogoll D., Melcher R., Richling E. (2017). Human intervention study to investigate the intestinal accessibility and bioavailability of anthocyanins from bilberries. Food Chem..

[53] Krga I., Milenkovic D. (2019). Anthocyanins: From Sources and Bioavailability to Cardiovascular-Health Benefits and Molecular Mechanisms of Action. J. Agric. Food Chem..

[54] Rȯhrig T., Kirsch V., Schipp D., Galan J., Richling E. (2019). Absorption of Anthocyanin Rutinosides after Consumption of a Blackcurrant (*Ribes nigrum* L.) Extract. J. Agric. Food Chem..

[55] Han F., Yang P., Wang H., Fernandes I., Mateus N., Liu Y. (2019). Digestion and absorption of red grape and wine anthocyanins through the gastrointestinal tract. Trends Food Sci. Technol..

[56] Kay C.D., Pereira-Caro G., Ludwig I.A., Clifford M.N., Crozier A. (2017). Anthocyanins and Flavanones Are More Bioavailable than Previously Perceived: A Review of Recent Evidence. Annu. Rev. Food Sci. Technol..

[57] Sandhu A.K., Miller M.G., Thangthaeng N., Scott T.M., Shukitt-Hale B., Edirisinghe I., Burton-Freeman B. (2018). Metabolic fate of strawberry polyphenols after chronic intake in healthy older adults. Food Funct..

[58] Czank C., Cassidy A., Zhang Q., Morrison D.J., Preston T., Kroon P.A., Botting N.P., Kay C.D. (2013). Human metabolism and elimination of the anthocyanin, cyanidin-3-glucoside: A 13C-tracer study. Am. J. Clin. Nutr..

[59] De Ferrars R.M., Czank C., Zhang Q., Botting N.P., Kroon P.A., Cassidy A., Kay C.D. (2014). The pharmacokinetics of anthocyanins and their metabolites in humans. Br. J. Pharmacol..

[60] Xie L., Lee S.G., Vance T.M., Wang Y., Kim B., Lee J.Y., Chun O.K., Bolling B.W. (2016). Bioavailability of anthocyanins and colonic polyphenol metabolites following consumption of aronia berry extract. Food Chem..

[61] Bresciani L., Angelino D., Vivas E.I., Kerby R.L., García-Viguera C., Del Rio D., Rey F.E., Mena P. (2020). Differential Catabolism of an Anthocyanin-Rich Elderberry Extract by Three Gut Microbiota Bacterial Species. J. Agric. Food Chem..

[62] Fornasaro S., Ziberna L., Gasperotti M., Tramer F., Vrhovšek U., Mattivi F., Passamonti S. (2016). Determination of cyanidin 3-glucoside in rat brain, liver and kidneys by UPLC/MS-MS and its application to a short-term pharmacokinetic study. Sci. Rep..

[63] Sandoval-Ramírez B.A., Catalán Ú., Fernández-Castillejo S., Rubio L., Macia A., Sola R. (2018). Anthocyanin tissue bioavailability in animals: Possible implications for human health: A systematic review. J. Agric. Food Chem..

[64] Marczylo T.H., Cooke D., Brown K., Steward W.P., Gescher A.J. (2009). Pharmacokinetics and Metabolism of the Putative Cancer Chemopreventive Agent Cyanidin-3-Glucoside in Mice. Cancer Chemother. Pharmacol..

[65] Kirakosyan A., Seymour E.M., Wolforth J., McNish R., Kaufman P.B., Bolling S.F. (2015). Tissue Bioavailability of Anthocyanins from Whole Tart Cherry in Healthy Rats. Food Chem..

[66] Halliwell B., Rafter J., Jenner A. (2005). Health promotion by flavonoids, tocopherols, tocotrienols, and other phenols: Direct or indirect effects? Antioxidant or not?. Am. J. Clin. Nutr..

[67] Halliwell B., Whiteman M. (2004). Measuring reactive species and oxidative damage in vivo and in cell culture: How should you do it and what do the results mean?. Br. J. Pharmacol..

[68] Tang S.Y., Halliwell B. (2010). Medicinal plants and antioxidants: What do we learn from cell culture and Caenorhabditis elegans studies?. Biochem. Biophys. Res. Commun..

[69] Apak R., Özyürek M., Güçlü K., Çapanoğlu E. (2016). Antioxidant Activity/Capacity Measurement. 1. Classification, Physicochemical Principles, Mechanisms, and Electron Transfer (ET)-Based Assays. J. Agric. Food Chem..

[70] Gulcin I. (2020). Antioxidants and antioxidant methods: An updated overview. Arch. Toxicol..

[71] Rahman M.M., Ichiyanagi T., Komiyama T., Sato S., Konishi T. (2008). Effects of Anthocyanins on Psychological Stress-Induced Oxidative Stress and Neurotransmitter Status. J. Agric. Food Chem..

[72] Khan M.S., Ali T., Kim M.W., Jo M.H., Chung J.I., Kim M.O. (2019). Anthocyanins Improve Hippocampus-Dependent Memory Function and Prevent Neurodegeneration via JNK/Akt/GSK3β Signaling in LPS-Treated Adult Mice. Mol. Neurobiol..

[73] Khan M.S., Ali T., Kim M.W., Jo M.H., Jo M.G., Badshah H., Kim M.O. (2016). Anthocyanins Protect Against LPS-induced Oxidative Stress-Mediated Neuroinflammation and Neurodegeneration in the Adult Mouse Cortex. Neurochem. Int..

[74] Rehman S.U., Shah S.A., Ali T., Chung J.I., Kim M.O. (2017). Anthocyanins Reversed D-Galactose-Induced Oxidative Stress and Neuroinflammation Mediated Cognitive Impairment in Adult Rats. Mol. Neurobiol..

[75] Ali T., Kim T., Rehman S.U., Khan M.S., Amin F.U., Khan M., Ikram M., Kim M.O. (2018). Natural Dietary Supplementation of Anthocyanins via PI3K/Akt/Nrf2/HO-1 Pathways Mitigate Oxidative Stress, Neurodegeneration, and Memory Impairment in a Mouse Model of Alzheimer’s Disease. Mol. Neurobiol..

[76] Qin L., Zhang J., Qin M. (2013). Protective Effect of Cyanidin 3-O-glucoside on Beta-Amyloid Peptide-Induced Cognitive Impairment in Rats. Neurosci. Lett..

[77] Wu X.L., Li X.X., Jis S.L., Gao Z.L., Lu Z., Dai X.L., Sun Y.X. (2017). Memory Enhancing and Antioxidant Activities of Lycium ruthenicum Murray Anthocyanin Extracts in an Aβ 42-Induced Rat Model of Dementia. Xiandai Shipin Keji.

[78] Chen S., Zhou H., Zhang G., Meng J., Deng K., Zhou W., Wang H., Wang Z., Hu N., Suo Y. (2019). Anthocyanins From Lycium Ruthenicum Murr. Ameliorated d-Galactose-Induced Memory Impairment, Oxidative Stress, and Neuroinflammation in Adult Rats. J. Agric. Food Chem..

[79] Lu X., Zhou Y., Wu T., Hao L. (2014). Ameliorative Effect of Black Rice Anthocyanin on Senescent Mice Induced by D-galactose. Food Funct..

[80] Wei J., Zhang G., Zhang X., Xu D., Gao J., Fan J., Zhou Z. (2017). Anthocyanins From Black Chokeberry (Aroniamelanocarpa Elliot) Delayed Aging-Related Degenerative Changes of Brain. J. Agric. Food Chem..

[81] Pacheco S.M., Soares M.S.P., Gutierres J.M., Gerzson M.F.B., Carvalho F.B., Azambuja J.H., Schetinger M.R.C., Stefanello F.M., Spanevello R.M. (2018). Anthocyanins as a Potential Pharmacological Agent to Manage Memory Deficit, Oxidative Stress and Alterations in Ion Pump Activity Induced by Experimental Sporadic Dementia of Alzheimer’s Type. J. Nutr. Biochem..

[82] Roghani M., Niknam A., Jalali-Nadoushan M.-R., Kiasalari Z., Khalili M., Baluchnejadmojarad T. (2010). Oral Pelargonidin Exerts Dose-Dependent Neuroprotection in 6-hydroxydopamine Rat Model of Hemi-Parkinsonism. Brain Res. Bull..

[83] Min J., Yu S.-W., Baek S.-H., Nair K.M., Bae O.-N., Bhatt A., Kassab M., Nair M.G., Majid A. (2011). Neuroprotective Effect of cyanidin-3-O-glucoside Anthocyanin in Mice With Focal Cerebral Ischemia. Neurosci. Lett..

[84] Skemiene K., Pampuscenko K., Rekuviene E., Borutaite V. (2020). Protective Effects of Anthocyanins against Brain Ischemic Damage. J. Bioenerg. Biomembr..

[85] Brader L., Overgaard A., Christensen L.P., Jeppesen P.B., Hermansen K. (2013). Polyphenol-Rich Bilberry Ameliorates Total Cholesterol and LDL-Cholesterol when Implemented in the Diet of Zucker Diabetic Fatty Rats. Rev. Diabet. Stud..

[86] Toufektsian M.C., de Lorgeril M., Nagy N., Salen P., Donati M.B., Giordano L., Mock H.P., Peterek S., Matros A., Petroni K. (2008). Chronic Dietary Intake of Plant-Derived Anthocyanins Protects the Rat Heart Against Ischemia-Reperfusion Injury. J. Nutr..

[87] Ziberna L., Lunder M., Moze S., Vanzo A., Tramer F., Passamonti S., Drevensek G. (2010). Acute Cardioprotective and Cardiotoxic Effects of Bilberry Anthocyanins in Ischemia-Reperfusion Injury: Beyond Concentration-Dependent Antioxidant Activity. Cardiovasc. Toxicol..

[88] Li F., Lang F., Wang Y., Zhai C., Zhang C., Zhang L., Hao E. (2018). Cyanidin ameliorates endotoxin-induced myocardial toxicity by modulating inflammation and oxidative stress through mitochondria and other factors. Food Chem. Toxicol..

[89] Skemiene K., Jablonskiene G., Liobikas J., Borutaite V. (2013). Protecting the Heart Against Ischemia/Reperfusion-Induced Necrosis and Apoptosis: The Effect of Anthocyanins. Medicina (Kaunas).

[90] Skemiene K., Liobikas J., Borutaite V. (2015). Anthocyanins as Substrates for Mitochondrial Complex I—Protective Effect against Heart Ischemic Injury. FEBS J..

[91] Arjinajarn P., Chueakula N., Pongchaidecha A., Jaikumkao K., Chatsudthipong V., Mahatheeranont S., Norkaew O., Chattipakorn N., Lungkaphin A. (2017). Anthocyanin-rich Riceberry Bran Extract Attenuates Gentamicin-Induced Hepatotoxicity by Reducing Oxidative Stress, Inflammation and Apoptosis in Rats. Biomed. Pharmacother..

[92] Hou F., Zhang R., Zhang M., Su D., Wei Z., Deng Y., Zhang Y., Chi J., Tang X. (2013). Hepatoprotective and Antioxidant Activity of Anthocyanins in Black Rice Bran on Carbon Tetrachloride-Induced Liver Injury in Mice. J. Funct. Foods.

[93] Chen J., Sun H., Sun A., Lin Q., Wang Y., Tao X. (2012). Studies of the Protective Effect and Antioxidant Mechanism of Blueberry Anthocyanins in a CC14-Induced Liver Injury Model in Mice. Food Agric. Immunol..

[94] Cho B.O., Ryu H.W., Jin C.H., Choi D.S., Kang S.Y., Kim D.S., Byun M.-W., Jeong I.Y. (2011). Blackberry Extract Attenuates Oxidative Stress through Up-Regulation of Nrf2-dependent Antioxidant Enzymes in Carbon Tetrachloride-Treated Rats. J. Agric. Food Chem..

[95] Sun J., Wu Y., Long C., He P., Gu J., Yang L., Liang Y., Wang Y. (2018). Anthocyanins Isolated From Blueberry Ameliorates CCl4 Induced Liver Fibrosis by Modulation of Oxidative Stress, Inflammation and Stellate Cell Activation in Mice. Food Chem. Toxicol..

[96] Zuo A., Wang S., Liu L., Yao Y., Guo J. (2019). Understanding the Effect of Anthocyanin Extracted from *Lonicera caerulea* L. On Alcoholic Hepatosteatosis. Biomed. Pharmacother..

[97] Wu S., Yano S., Hisanaga A., He X., He J., Sakao K., Hou D.-X. (2017). Polyphenols from *Lonicera caerulea* L. Berry Attenuate Experimental Nonalcoholic Steatohepatitis by Inhibiting Proinflammatory Cytokines Productions and Lipid Peroxidation. Mol. Nutr. Food Res..

[98] Prokop J., Anzenbacher P., Mrkvicová E., Pavlata L., Zapletalová I., Šťastník O., Martinek P., Kosina P., Anzenbacherová E. (2018). In Vivo Evaluation of Effect of Anthocyanin-Rich Wheat on Rat Liver Microsomal Drug-Metabolizing Cytochromes P450 and on Biochemical and Antioxidant Parameters in Rats. Food Chem. Toxicol..

[99] Popović D., Kocić G., Katić V., Jović Z., Zarubica A., Janković Veličković L., Nikolić V., Jović A., Kundalić B., Rakić V. (2019). Protective Effects of Anthocyanins From Bilberry Extract in Rats Exposed to Nephrotoxic Effects of Carbon Tetrachloride. Chem. Biol. Interact..

[100] Yarijani Z.M., Najafi H., Shackebaei D., Madani S.H., Modarresi M., Jassemi S.V. (2019). Amelioration of Renal and Hepatic Function, Oxidative Stress, Inflammation and Histopathologic Damages by Malva Sylvestris Extract in Gentamicin Induced Renal Toxicity. Biomed. Pharmacother..

[101] Veljković M., Pavlović D.R., Stojiljković N., Ilić S., Jovanović I., Poklar Ulrih N., Rakić V., Veličković L., Sokolović D. (2017). Bilberry: Chemical Profiling, in vitro and in vivo Antioxidant Activity and Nephroprotective Effect Against Gentamicin Toxicity in Rats. Phytother. Res..

[102] Qi Z.-L., Wang Z., Li W., Hou J.-G., Liu Y., Li X.-D., Li H.-P., Wang Y.-P. (2017). Nephroprotective Effects of Anthocyanin from the Fruits of Panax Ginseng (GFA) on Cisplatin-Induced Acute Kidney Injury in Mice. Phytother. Res..

[103] Lee I.-C., Bae J.-S. (2019). Pelargonidin Protects Against Renal Injury in a Mouse Model of Sepsis. J. Med. Food.

[104] Suda I., Ishikawa F., Hatakeyama M., Miyawaki M., Kudo T., Hirano K., Ito A., Yamakawa O., Horiuchi S. (2008). Intake of Purple Sweet Potato Beverage Affects on Serum Hepatic Biomarker Levels of Healthy Adult Men With Borderline Hepatitis. Eur. J. Clin. Nutr..

[105] Gao X., Cassidy A., Schwarzschild M.A., Rimm E.B., Ascherio A. (2012). Habitual intake of dietary flavonoids and risk of Parkinson disease. Neurology.

[106] Valenti L., Riso P., Mazzocchi A., Porrini M., Fargion S., Agostoni C. (2013). Dietary Anthocyanins as Nutritional Therapy for Nonalcoholic Fatty Liver Disease. Oxid. Med. Cell. Longev..

[107] Vendrame S., Del Bo C., Ciappellano S., Riso P., Klimis-Zacas D. (2016). Berry Fruit Consumption and Metabolic Syndrome. Antioxidants.

[108] Oki T., Kano M., Ishikawa F., Goto K., Watanabe O., Suda I. (2017). Double-blind, Placebo-Controlled Pilot Trial of Anthocyanin-Rich Purple Sweet Potato Beverage on Serum Hepatic Biomarker Levels in Healthy Caucasians With Borderline Hepatitis. Eur. J. Clin. Nutr..

[109] Fairlie-Jones L., Davison K., Fromentin E., Hill A.M. (2017). The Effect of Anthocyanin-Rich Foods or Extracts on Vascular Function in Adults: A Systematic Review and Meta-Analysis of Randomised Controlled Trials. Nutrients.

[110] Godos J., Vitale M., Micek A., Ray S., Martini D., Del Rio D., Riccardi G., Galvano F., Grosso G. (2019). Dietary Polyphenol Intake, Blood Pressure, and Hypertension: A Systematic Review and Meta-Analysis of Observational Studies. Antioxidants.

[111] Ullah R., Khan M., Shah S.A., Saeed K., Kim M.O. (2019). Natural Antioxidant Anthocyanins-A Hidden Therapeutic Candidate in Metabolic Disorders With Major Focus in Neurodegeneration. Nutrients.

[112] Winter A.N., Bickford P.C. (2019). Anthocyanins and Their Metabolites as Therapeutic Agents for Neurodegenerative Disease. Antioxidants.

[113] Krikorian R., Kalt W., McDonald J.E., Shidler M.D., Summer S.S., Stein A.L. (2020). Cognitive performance in relation to urinary anthocyanins and their flavonoid-based products following blueberry supplementation in older adults at risk for dementia. J. Funct. Foods.

[114] Cásedas G., Les F., López V. (2020). Anthocyanins: Plant Pigments, Food Ingredients or Therapeutic Agents for the CNS? A Mini-Review Focused on Clinical Trials. Curr. Pharm. Des..

[115] Danielewski M., Matuszewska A., Nowak B., Kucharska A.Z., Sozański T. (2020). The Effects of Natural Iridoids and Anthocyanins on Selected Parameters of Liver and Cardiovascular System Functions. Oxid. Med. Cell. Longev..

[116] Davinelli S., Bertoglio J.C., Zarrelli A., Pina R., Scapagnini G. (2015). A Randomized Clinical Trial Evaluating the Efficacy of an Anthocyanin-Maqui Berry Extract (Delphinol^®^) on Oxidative Stress Biomarkers. J. Am. Coll. Nutr..

[117] Toaldo I.M., Cruz F.A., de Lima Alves T., de Gois J.S., Borges D.L.G., Cunha H.P., da Silva E.L., Bordignon-Luiz M.T. (2015). Bioactive Potential of Vitis Labrusca L. Grape Juices from the Southern Region of Brazil: Phenolic and Elemental Composition and Effect on Lipid Peroxidation in Healthy Subjects. Food Chem..

[118] Bialasiewicz P., Prymont-Przyminska A., Zwolinska A., Sarniak A., Wlodarczyk A., Krol M., Markowski J., Rutkowski K.P., Nowak D. (2018). Sour Cherries but Not Apples Added to the Regular Diet Decrease Resting and fMLP-Stimulated Chemiluminescence of Fasting Whole Blood in Healthy Subjects. J. Am. Coll. Nutr..

[119] Del Bó C., Riso P., Campolo J., Møller P., Loft S., Klimis-Zacas D., Brambilla A., Rizzolo A., Porrini M. (2013). A Single Portion of Blueberry (*Vaccinium corymbosum* L) Improves Protection against DNA Damage but Not Vascular Function in Healthy Male Volunteers. Nutr. Res..

[120] Kropat C., Mueller D., Boettler U., Zimmermann K., Heiss E.H., Dirsch V.M., Rogoll D., Melcher R., Richling E., Marko D. (2013). Modulation of Nrf2-dependent Gene Transcription by Bilberry Anthocyanins in vivo. Mol. Nutr. Food Res..

[121] Erlund I., Koli R., Alfthan G., Marniemi J., Puukka P., Mustonen P., Mattila P., Jula A. (2008). Favorable Effects of Berry Consumption on Platelet Function, Blood Pressure, and HDL Cholesterol. Am. J. Clin. Nutr..

[122] Cassidy A., Rogers G., Peterson J.J., Dwyer J.T., Lin H., Jacques P.F. (2015). Higher Dietary Anthocyanin and Flavonol Intakes Are Associated With Anti-Inflammatory Effects in a Population of US Adults. Am. J. Clin. Nutr..

[123] Natella F., Macone A., Ramberti A., Forte M., Mattivi F., Matarese R.M., Scaccini C. (2011). Red Wine Prevents the Postprandial Increase in Plasma Cholesterol Oxidation Products: A Pilot Study. Br. J. Nutr..

[124] Estruch R., Sacanella E., Mota F., Chiva-Blanch G., Antúnez E., Casals E., Deulofeu R., Rotilio D., Andres-Lacueva C., Lamuela-Raventos R.M. (2011). Moderate Consumption of Red Wine, but Not Gin, Decreases Erythrocyte Superoxide Dismutase Activity: A Randomised Cross-Over Trial. Nutr. Metab. Cardiovasc. Dis..

[125] Zhu Y., Ling W., Guo H., Song F., Ye Q., Zou T., Li D., Zhang Y., Li G., Xiao Y. (2013). Anti-inflammatory Effect of Purified Dietary Anthocyanin in Adults With Hypercholesterolemia: A Randomized Controlled Trial. Nutr. Metab. Cardiovasc. Dis..

[126] Curtis P.J., van der Velpen V., Berends L., Jennings A., Feelisch M., Umpleby A.M., Evans M., Fernandez B.O., Meiss M.S., Minnion M. (2019). Blueberries Improve Biomarkers of Cardiometabolic Function in Participants With Metabolic Syndrome-Results From a 6-month, Double-Blind, Randomized Controlled Trial. Am. J. Clin. Nutr..

[127] Zhang P.-W., Chen F.-X., Li D., Ling W.-H., Guo H.-H. (2015). A CONSORT-compliant, Randomized, Double-Blind, Placebo-Controlled Pilot Trial of Purified Anthocyanin in Patients with Nonalcoholic Fatty Liver Disease. Medicine (Baltimore).

[128] Guo H., Zhong R., Liu Y., Jiang X., Tang X., Li Z., Xia M., Ling W. (2014). Effects of Bayberry Juice on Inflammatory and Apoptotic Markers in Young Adults With Features of Non-Alcoholic Fatty Liver Disease. Nutrition.

[129] Spormann T.M., Albert F.W., Rath T., Dietrich H., Will F., Stockis J.-P., Eisenbrand G., Janzowski C. (2008). Anthocyanin/polyphenolic-rich Fruit Juice Reduces Oxidative Cell Damage in an Intervention Study with Patients on Hemodialysis. Cancer Epidemiol. Biomark. Prev..

[130] Castilla P., Dávalos A., Teruel J.L., Cerrato F., Fernández-Lucas M., Merino J.L., Sánchez-Martín C.C., Ortuño J., Lasunción M.A. (2008). Comparative Effects of Dietary Supplementation With Red Grape Juice and Vitamin E on Production of Superoxide by Circulating Neutrophil NADPH Oxidase in Hemodialysis Patients. Am. J. Clin. Nutr..

